# ILC1s and ILC3s Exhibit Inflammatory Phenotype in Periodontal Ligament of Periodontitis Patients

**DOI:** 10.3389/fimmu.2021.708678

**Published:** 2021-07-26

**Authors:** Changyi Li, Jianyue Liu, Jie Pan, Yuhui Wang, Lei Shen, Yan Xu

**Affiliations:** ^1^ Department of Orthodontics, Shanghai Stomatological Hospital, Fudan University, Shanghai, China; ^2^ Shanghai Key Laboratory of Craniomaxillofacial Development and Diseases, Fudan University, Shanghai, China; ^3^ Shanghai Institute of Immunology, Shanghai Jiao Tong University School of Medicine, Shanghai, China

**Keywords:** innate lymphoid cells, periodontitis, periodontal ligament, inflammation, IL-17A, IFN-γ

## Abstract

Innate lymphoid cells (ILCs) are emerging as important players in inflammatory diseases. The oral mucosal barrier harbors all ILC subsets, but how these cells regulate the immune responses in periodontal ligament tissue during periodontitis remains undefined. Here, we show that total ILCs are markedly increased in periodontal ligament of periodontitis patients compared with healthy controls. Among them, ILC1s and ILC3s, particularly NKp44^+^ILC3 subset, are the predominant subsets accumulated in the periodontal ligament. Remarkably, ILC1s and ILC3s from periodontitis patients produce more IL-17A and IFN-γ than that from healthy controls. Collectively, our results highlight the role of ILCs in regulating oral immunity and periodontal ligament inflammation and provide insights into targeting ILCs for the treatment of periodontitis.

## Introduction

Periodontitis is a common chronic inflammatory disease characterized by oral mucosal tissue destructive inflammation, which leads to periodontal ligament damage, loss of supporting bone, and ultimately, tooth loss (1, 2). Microbial dysbiosis is thought to be the trigger of the oral soft tissue inflammation and current standard of care largely rely on the removal of microbial biofilm by mechanical removal or antibiotics (3). Nevertheless, it is not curative. Therefore, there is an urgent need to understand the pathogenesis of the destructive tissue inflammation.

Innate lymphoid cells (ILCs) are a recently identified family of tissue-resident lymphocytes that lack adaptive antigen receptors. Based on the expression of signature cytokines and transcription factors, ILCs are divided into three major groups: T-bet^+^ ILC1s produce interferon-γ (IFN-γ); GATA3^+^ ILC2s secrete interleukin-5 (IL-5), IL-13, and amphiregulin; and RORγt^+^ ILC3s produce IL-17A and IL-22. ILC3s can be further divided into natural cytotoxicity receptor (NCR)^+^ ILC3s, NCR^-^ILC3s and lymphoid tissue inducer (LTi) cells (4, 5). ILCs mainly reside at mucosal barrier surfaces that are able to provide an immediate immune response with rapid cytokines secretion in response to environmental stimuli (6). Thus, ILCs play important roles in regulating inflammation, immunity, tissue repair, and homeostasis.

Increasing numbers of studies have implicated that ILCs are critical regulators in multiple inflammatory diseases (7). ILC2s promote airway inflammation through secreting type 2 cytokines of IL-4, IL-5, and IL-13 and contribute to the pathogenesis of allergic asthma. Increased ILC2s are found in the blood and BALF of patients with asthma (8, 9). ILC3s and ILC1s are the abundant populations of ILCs in the human gut and have been extensively studied in inflammatory bowel disease (IBD) (10). The number of IFN-γ–producing ILC1s and IL-17A–producing NCR^-^ ILC3s are significantly increased in the inflamed intestinal mucosa of patients with Crohn’s disease (11). However, the increase of IFN-γ–producing ILC1s is accompanied by a large decrease in the number of NCR^+^ ILC3s, which is thought to be due to the plasticity between ILC3s and ILC1s (12, 13). Studies from both mice and human have demonstrated that NCR^+^ ILC3s can convert into IFN-γ–producing ILC1s (14, 15). ILC3s have also been shown to contribute to the skin inflammation of psoriasis (16).

As one of the important barrier sites, the oral mucosa is constantly exposed to a plethora of environmental stimuli, including microbes, food antigens, and airborne particles (17). Thus, immune responses in oral barrier need to be tightly controlled to maintain homeostatic balance between tolerance to commensals and immunity to pathogens (18). Oral mucosa harbors all ILC subsets (19–21). Two recent studies have shown that ILCs are involved in periodontitis (20, 21).One shows that all ILC subsets are increased in periodontitis patients, with a marked effect on ILC2s (20). Another study shows ILC1s are the predominant subset with RANKL expression in gingivitis and periodontitis (21). However, whether ILCs have any functional abnormalities in periodontitis remain largely unknown. In this study, we show ILCs in periodontal ligament are markedly increased in periodontitis patients compared with healthy controls. Among various ILCs, ILC1s and ILC3s exhibit increased numbers in periodontal ligament and enhanced production of inflammatory cytokines in periodontitis. Thus, our results highlight the role of ILCs in regulating oral immunity and periodontal ligament inflammation and provide insights into targeting ILCs for the treatment of periodontitis.

## Materials and Methods

### Study Approval

The study was approved by the ethical review board of Shanghai Stomatological Hospital, Fudan University (2021-008). All individuals included in this study were >18 years of age. All samples came from separate individuals, and each individual only donated one sample each. Written informed consent from all participants was obtained prior to the study.

### Sample Collection

All participants were in good general health, non-smokers, non-diabetes, or other chronic inflammatory disease and had not taken antibiotics or anti-inflammatory drugs within the past 3 months (**Table S1**). Twenty-five teeth of healthy participants were obtained from individuals undergoing maxillofacial surgery of third molar extraction at the Department of Oral and Maxillofacial Surgery, Shanghai Stomatological Hospital, Fudan University. The inclusion criteria were as follows: (i) tooth displaying no bleeding on probing (BOP), (ii) no radiographic bone loss, and (iii) no periodontal pockets exceeding >3 mm or clinical attachment loss. Twenty-six teeth from periodontitis patients were obtained from patients undergoing teeth extraction with poor prognosis due to severe alveolar bone loss and periodontal damage. The inclusion criteria were as follows: (i) radiographic bone loss>60% of the root, (ii) periodontal pockets exceeding >6 mm or clinical attachment loss.

### Tissue Processing and Preparation

The collected tissue samples (teeth) were immediately put in 5 ml of RPMI 1640 medium containing 10% FBS on ice and subject for process within one hour. Each sample was washed with PBS to remove visible blood. Subsequently, gingiva tissues attached to the teeth were removed with razor blade. The periodontal ligament tissue on the root surface was collected using a scalpel blade and recorded for its weight. Then the tissues were digested in a 15-ml centrifuge tube with 2-ml RPMI 1640 medium containing 2% FBS, 2 mg/ml of Collagenase Type II (Worthington Biochemical), and 1 mg/ml of DNAse Type I (Sigma-Aldrich), for 1 h at 37°C. The digested tissues were passed through a 70μm cell strainer after vigorous shaking for 1 min, followed by centrifugation at 500*g* for 5 min. Red blood cells were lysed with 1ml ACK lysis buffer for 2 min, then passed through a 40-μm cell strainer to obtain the single-cell suspensions of periodontal ligaments. The cells were then stained with trypan blue and counted using a cell counting plate to record the total cell numbers of each sample.

### Flow Cytometry

Single-cell suspensions isolated from periodontal ligaments were first stained with fixable viability stain 520 (BD Biosciences) for 10 min to remove the dead cells. Anti-CD16/32 antibody (Biolegend) was used to block non-specific binding to Fc receptors before surface staining. Surface markers included the following antibodies: anti-CD3-FITC (UCHT1) (BioLegend), anti-CD19-FITC (HIB19) (BioLegend), anti-CD14-FITC (M5E2) (BioLegend), anti-CD16-FITC (eBioCB16) (eBioscience), anti-CD1a-FITC (HI149) (eBioscience), anti-CD123-FITC (6H6) (BioLegend), anti-CD303-FITC (201A) (BioLegend), anti-FcϵR1α-FITC (AER-37) (eBioscience), anti-CD34-FITC (581) (BioLegend), anti-CD94-FITC (DX22) (BioLegend), anti-CD127-BV786 (eBioRDR5) (Invitrogen), anti-CD45-AF700 (HI30) (BioLegend), anti-CD117-BV421 (104D2) (BioLegend), anti-CRTH2-PE-CF594 (BM16) (BioLegend), anti-NKp44-BB700 (P44-8) (BioLegend), and anti-CD161-BV605 (HP-3G10) (BioLegend). After 30 min of incubation, cells were washed with PBS, centrifuged at 400*g* for 5 min, the supernatant was discarded, then the pellet was resuspended in 200 µl PBS.

For intracellular cytokines staining, total cells were stimulated with 50 ng/ml PMA (Sigma-Aldrich) plus 500 ng/ml ionomycin (Sigma-Aldrich) or 50 ng/ml human IL-23 (R&D systems) plus 50 ng/ml human IL-1β (R&D systems) or 50 ng/ml human IL-12 (Peprotech), in the presence of 5 μg/ml brefeldin A (BioLegend) for 6 h. The cells were stained with surface markers described above, then permeabilized and stained with intracellular antibodies anti-IL-17A-PE-Cy7 (BL168) (BioLegend), anti-IFN-γ-BUV563 (B27) (BD Biosciences), anti-T-bet-APC (4B10) (eBioscience), and anti-RORγt-PE (AFKJS-9) (eBioscience), using the Foxp3/Transcription Factor Staining Buffer Set (Invitrogen). All data were collected on a BD FACSymphony A3 (BD Biosciences). The analysis of flow cytometry data were carried out using FlowJo™ v10.7 software.

### Statistical Analysis

Unless otherwise indicated, all statistical analyses were performed using Graphpad Prism 8 software. All the data were first tested for normal distribution. Data with normal distribution, statistical significance was evaluated by two-tailed unpaired Student’s t-test. Data without normal distribution and statistical significance was calculated by nonparametric Mann-Whitney test. P values less than 0.05 were considered to be statistically significant. Data were presented as means ± standard error of the mean (s.e.m.).

## Results

### ILCs Are Increased in Periodontal Ligament of Periodontitis Patients

**Figure 1:**
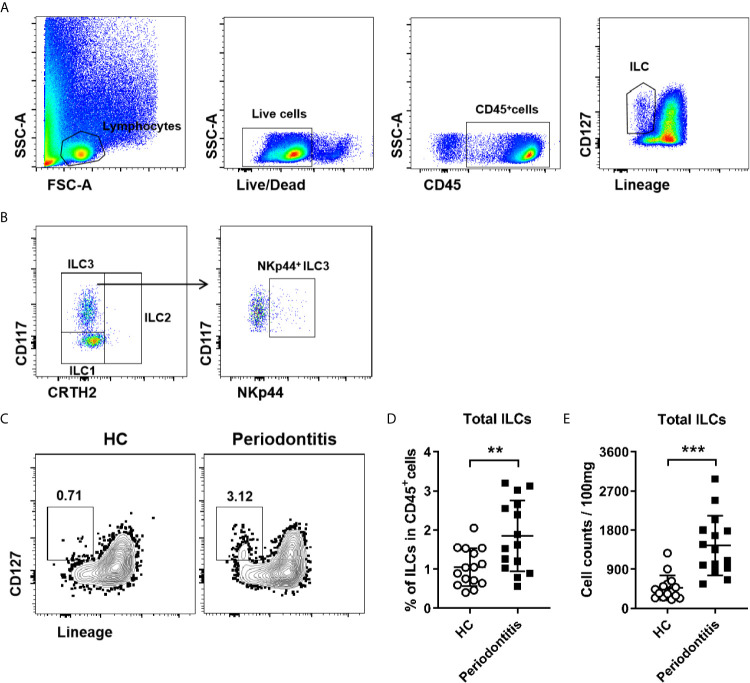
Increased ILCs in periodontal ligament tissue from periodontitis patients. **(A)** Gating strategy for total ILCs in human periodontal ligament tissue. Lineage markers include CD3, CD19, CD94, CD14, CD16, CD1a, CD303, CD123, FcϵR1α, and CD34. **(B)** Gating strategy for ILCs subsets in human periodontal ligament tissue. **(C)** Representative flow cytometry analysis of total ILCs in periodontal ligament tissue from healthy individuals (left) and periodontitis patients (right). **(D)** Frequency (in CD45^+^ immune cells) and **(E)** absolute cell numbers (per 100 mg tissue) of total ILCs in periodontal ligament tissue from healthy controls and periodontitis patients. n = 15 for each group. **p < 0.01, ***p < 0.001.

Periodontal ligament tissues were collected from 25 healthy controls and 26 periodontitis patients (**Table S1**). ILCs residing in periodontal ligament were analyzed by flow cytometry. The gating strategy of total ILCs and ILC subsets was shown in **Figures 1A, B**. Total ILCs are characterized by the expression of CD45 and CD127 but lack of lineage markers, including T cells (CD3), B cells (CD19), NK cells (CD16 and CD94), monocytes (CD14 and CD16), dendritic cells (CD1a, CD303 and CD123), granulocytes (FcϵR1α and CD123), mast cells (FcϵR1α), basophils (FcϵR1α), and hematopoietic stem cells (CD34) (22, 23). ILC2s are defined by the expression of CRTH2 (chemoattractant receptor-homologous molecule expressed on TH2 cells), ILC3s are classified by the expression of CD117. Human ILC3s are further divided into NKp44^+^ and NKp44- subsets. However, there is no specific surface marker for ILC1s, which are currently described as CRTH2^−^CD117^−^ILCs (24). Here, we first observed increased lymphocytes infiltration in periodontal ligament of periodontitis patients (**Figures S1A, B**), and the percentage of total ILCs in periodontal ligament was markedly increased in periodontitis patients compared with healthy individuals (**Figures 1C, D**). Consistently, the absolute cell number of total ILCs in periodontitis patients was higher than that in healthy controls (**Figure 1E**). Together, our data suggest the presence of ILCs in periodontal ligament tissue and increased ILCs during periodontitis.

### ILC1s and ILC3s Are Accumulated in Periodontal Ligament Tissue of Periodontitis Patients

**Figure 2:**
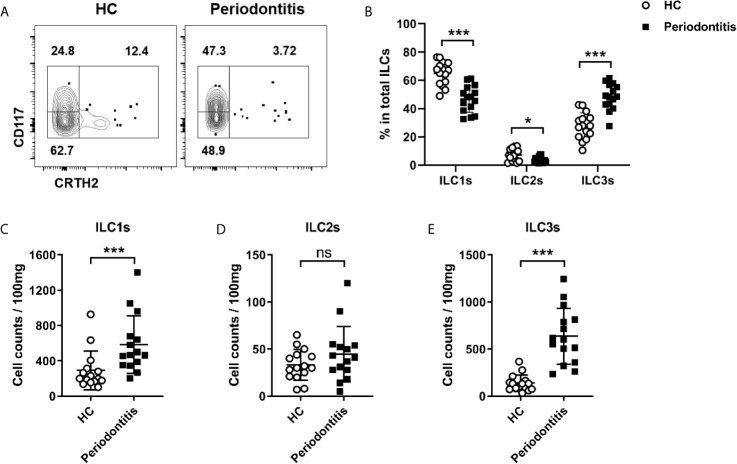
Distribution of distinct ILCs subsets in periodontal ligament of healthy controls and periodontitis patients. **(A)** Representative flow cytometry analysis of ILC1s, ILC2s, and ILC3s in periodontal ligament from healthy individuals (left) and periodontitis patients (right). **(B)** Frequencies (in total ILCs) of ILC1s, ILC2s, and ILC3s in periodontal ligament from healthy individuals and periodontitis patients. Absolute cell numbers (per 100 mg tissue) of **(C)** ILC1s, **(D)** ILC2s, and **(E)** ILC3s in periodontal ligament from healthy individuals and periodontitis patients. n=15 for each group. *p < 0.05, ***p < 0.001, ns, not significant.

ILCs are subdivided into three subsets: ILC1s, ILC2s, and ILC3s. Given that the total ILCs were increased in periodontal ligament of periodontitis, we next sought to determine which subset was affected mostly. Three ILC subsets were analyzed by flow cytometry in both healthy controls and periodontitis patients. ILC1s and ILC3s were the predominant subsets in periodontal ligament in both healthy controls and periodontitis patients, accounting for over 80% of the total ILCs. By contrast, the proportion of ILC2s was less than 20% of total ILCs (**Figure 2A**). Intriguingly, the percentage of ILC3s was significantly increased in periodontitis group compared with healthy controls, whereas the percentages of ILC1s and ILC2s were decreased correspondingly (**Figure 2B**). Because the total ILCs number was increased, we next analyzed the absolute cell counts for each subset to ensure the changes in periodontitis. Strikingly, ILC1s and ILC3s were accumulated in periodontitis patients (**Figures 2C, E**). Despite the percentage of ILC2s being lower in the periodontitis group, the absolute count of ILC2 did not show significant difference between healthy controls and periodontitis patients (**Figure 2D**). Collectively, these results demonstrate that ILC3s and ILC1s are accumulated in periodontal ligament of periodontitis patients, suggesting a role for these cells in the pathophysiology of periodontitis.

### NKp44^+^ILC3s Are Increased in the Periodontal Ligament of Periodontitis Patients

**Figure 3:**
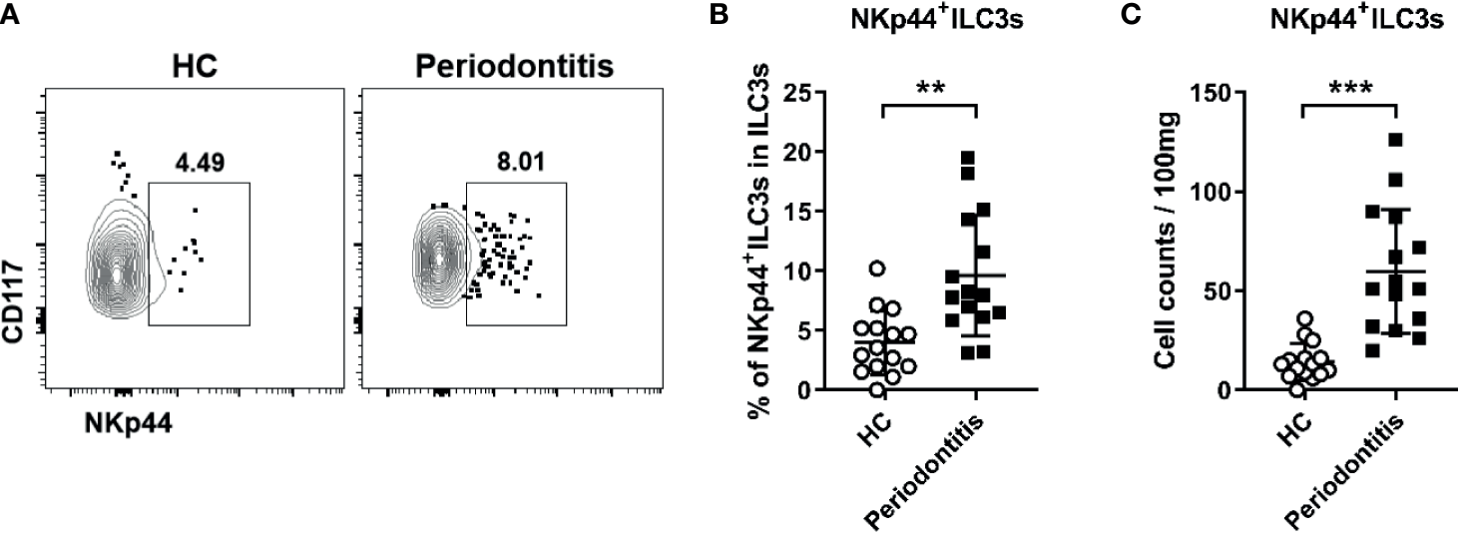
Increased NKp44^+^ ILC3s in periodontal ligament of periodontitis patients. **(A)** Representative flow cytometry analysis of NKp44^+^ ILC3s in periodontal ligament from healthy individuals (left) and periodontitis patients (right). **(B)** Frequency (in total ILC3s) and **(C)** absolute cell numbers (per 100 mg tissue) of NKp44^+^ ILC3s in periodontal ligament from healthy individuals and periodontitis patients. n = 15 for each group. **p < 0.01, ***p < 0.001.

In humans, ILC3s can be further subclassified as NKp44^+^ILC3s and NKp44^-^ILC3s. To determine whether the distribution of ILC3 subsets was altered in periodontitis, the proportion of ILC3 subsets in the periodontal ligament was analyzed by flow cytometry. Notably, NKp44^+^ILC3s accounted only for a small proportion of total ILC3s at approximately 5% (**Figure 3A**). Despite the scarcity, both the frequency and absolute count of NKp44^+^ILC3s in periodontitis patients were markedly increased compared with that in healthy controls (**Figures 3B, C**). These results raised a possibility that NKp44^+^ILC3s might play a pathogenic role in periodontitis.

### ILC1s and ILC3s Exhibit Inflammatory Phenotype With Enhanced IFN-γ and IL-17A Production in Periodontal Ligament of Periodontitis Patients

**Figure 4:**
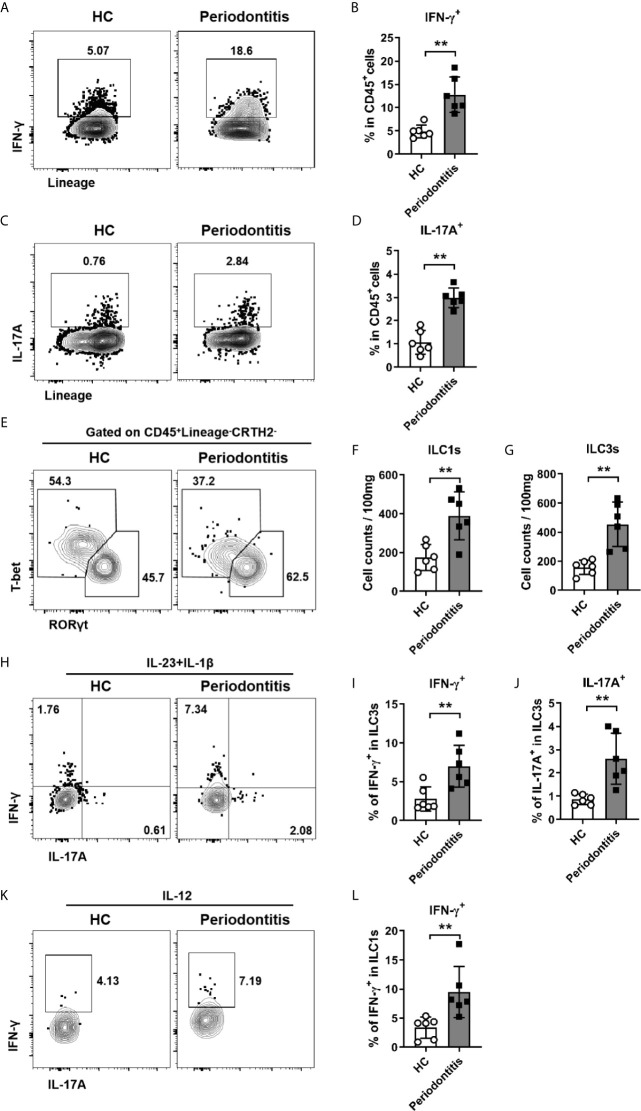
Enhanced production of IFN-γ and IL-17A by ILC1s/ILC3s in periodontal ligament of periodontitis patients. **(A)** Representative flow cytometry analysis of CD45^+^ immune cells stimulated with PMA plus ionomycin and stained for intracellular IFN-γ. **(B)** Frequencies of IFN-γ^+^ cells in CD45^+^ cells from healthy individuals and periodontitis patients. **(C)** Representative flow cytometry analysis of CD45^+^ immune cells stimulated with PMA plus ionomycin and stained for intracellular IL-17A. **(D)** Frequencies of IL-17A^+^ cells in CD45^+^ cells from healthy individuals and periodontitis patients. **(E)** Representative flow cytometry analysis of RORγt and T-bet in CD45^+^Lineage^-^CRTH2^-^ cells. Absolute cell numbers (per 100mg tissue) of **(F)** ILC1s (T-bet^+^) and **(G)** ILC3s (RORγt^+^) in periodontal ligament from healthy individuals and periodontitis patients. **(H)** Representative flow cytometry analysis of intracellular IL-17A and IFN-γ in ILC3s (gated on CD45^+^Lineage^-^CRTH2^-^RORγt^+^ cells) stimulated with IL-23 and IL-1β. Frequencies of **(I)** IFN-γ^+^ cells and **(J)** IL-17A^+^ cells in ILC3s from healthy individuals and periodontitis patients. **(K)** Representative flow cytometry analysis of intracellular IFN-γ in ILC1s (gated on CD45^+^Lineage^-^CRTH2^-^T-bet^+^ cells) stimulated with IL-12. **(L)** Frequencies of IFN-γ^+^ cells in ILC1s from healthy individuals and periodontitis patients. n = 6 for each group. **p < 0.01.

Given ILCs are an important source of IL-17A and IFN-γ, which have been shown to be associated with periodontitis susceptibility in a large quantity of clinical studies (11, 25–29), we next sought to determine whether ILCs from periodontitis patients could produce more inflammatory cytokines. Cells isolated from periodontal ligament were stimulated with PMA/ionomycin and followed by intracellular staining for cytokines production. We first examined IFN-γ and IL-17A production in total CD45^+^ immune cells. Both IFN-γ and IL-17A expression were dramatically increased in periodontitis patients (**Figures 4A–D**). Then the expression of IFN-γ and IL-17A by ILCs was analyzed. Notably, ILC3s cannot be separated from ILC1s due to the downregulation of CD117 after PMA and ionomycin stimulation (**Figures S2A–C**). Thus, we used transcription factors T-bet and RORγt to distinguish ILC1s and ILC3s (**Figure 4E**). Consistent with the surface staining results, the numbers of both ILC1s and ILC3s defined by transcription factors were increased in periodontitis group (**Figures 4F, G**). To mimic the physiological condition to activate ILCs, IL-23/IL-1β and IL-12 were used to stimulate ILC3s and ILC1s, respectively. Upon stimulation with IL-23 and IL-1β, the expression of IFN-γ and IL-17A by ILC3s was markedly enhanced in periodontitis patients compared to healthy controls (**Figures 4H–J**). Consistently, ILC1s from periodontitis patients produced more IFN-γ than that from healthy donors upon stimulation with IL-12 (**Figures 4K, L**). Collectively, these data suggest that ILC1s and ILC3s promote local inflammatory responses in periodontal ligament tissue of periodontitis through producing inflammatory cytokines of IFN-γ and IL-17A.

## Discussion

Innate lymphoid cells play critical roles in regulating immunity and inflammation, particularly at the mucosal barrier sites (6). Periodontitis is a common inflammatory disease in oral mucosal barrier that leads to immunopathology and destruction of supporting bone (1). However, the role of ILCs in periodontitis is poorly understood. Here, we have shown that both ILC1s and ILC3s are dramatically increased in periodontal ligament of periodontitis patients. Moreover, these cells exhibit an inflammatory phenotype indicated by enhanced production of IFN-γ and IL-17A in periodontitis. These data suggest ILC1s and ILC3s might promote the periodontal ligament inflammation and contribute to the pathogenesis of periodontitis.

Previous studies have shown that all ILCs subset are present in periodontal tissues from both gingivitis and periodontitis patients (20, 21). One study found that ILC1s are the major subset with ILC compartment in soft tissue from both gingivitis and periodontitis (21). While the other group showed that ILC2s are the predominant subset in periodontitis (20). The results from these two studies contradict each other. This might be because of the difference in samples collection. Soft tissues were used for the first study. The periodontal tissues were studied in the latter study, but they did not specify whether gingiva tissues or periodontal ligament tissues were used. In our study, we focused on the severe periodontitis patients who require surgery for tooth extraction. Periodontal ligament tissues were isolated and processed for subsequent cell analysis, which was thought to be more precise to reflect the local tissue-specific immune response during periodontitis. Periodontal ligament is the connective tissue that harbors both on tooth root and alveolar bone. When bacteria and LPS intrude the sulcular epithelium, the immune response is initiated in periodontal ligament that cause attachment loss, which forms periodontal pocket and alveolar bone loss. The immune response in periodontal ligament is the main cause of periodontal tissue destruction and alveolar bone loss.

The healthy individuals were included as control group. We chose a healthy third molar as a control. Although the mechanical load to the teeth maybe different from third molars, molars, or pre-molars. Biological mechanical load will do no harm to the periodontal tissue. Only occlusal trauma can lead periodontal tissue damage, which is excluded in our study. Furthermore, the construction and composition of periodontal ligament of molar and third molar is same, which can be used as healthy control group.

First, we found that ILC1s were the most abundant cell subset in periodontal ligament tissue from both healthy controls and periodontitis patients, accounting for about 50% of the total ILCs. By contrast, ILC2s were the least population of ILCs compartment account for less 10% of total ILCs. Then, we observed that total ILCs were dramatically increased in the periodontal ligament of periodontitis patients. Among them, the numbers of ILC1s and ILC3s were abnormally high in periodontal ligament tissues from periodontitis patients. These findings are consistent with the increased ILC1s and ILC3s in other inflammatory diseases, such as IBD and psoriasis.

Human ILC3s include NKp44^+^ILC3s and NKp44^-^ILC3s. NKp44^+^ILC3s are able to produce IL-17A, IL-22 and IFN-γ, whereas NKp44^-^ILC3s mainly produce IL-17A (30, 31). Previous reports have shown that NKp44^+^ILC3s are enriched in mucosal barrier tissues, such as intestinal tract, tonsils, and skin and are increased in inflammatory diseases, such as psoriasis and IBD (12, 30, 31). Despite NKp44^+^ILC3s are the largest population accounting for 70% of the total intestinal ILCs in the human gut (12, 14), they appeared to be a rare population in periodontal ligament accounting for less than 5% of the total ILCs. The difference in tissue distribution might reflect the influence of various tissue environment signals on the differentiation of ILC3 subsets. NKp44^+^ILC3s are considered as an inflammatory subset given their capability to produce IFN-γ and IL-17A. Increased NKp44^+^ILC3s have been shown in the skin lesions of psoriasis patients (16). Consistently, we observed that NKp44^+^ILC3s were increased as well as in inflamed periodontal ligament tissue. Together with the increased NKp44^+^ILC3s, the production of IFN-γ by ILC3s was enhanced as well. These data raised the possibility that increased IFN-γ might come from NKp44^+^ILC3s and contribute to the disease. These data indicate that ILC3s might play a pathogenic role in promoting the periodontal inflammation during periodontitis.

Previous reports have shown that inflammatory cytokines IL-17A and IFN-γ are implicated in driving the pathogenesis of periodontitis and consequent bone and tooth loss (32). IL-17A is increased in periodontitis and positively correlated with disease severity (33). IL-17A can promote osteoclastogenesis and bone resorption through activation osteoblasts to produce RANKL. In addition, IL-17A can synergize with other inflammatory cytokines to induce inflammatory responses, which result in destruction of periodontal tissues and alveolar bone (34). Similar to IL-17A, IFN-γ is also increased in periodontitis patients (35). IFN-γ has been shown not only to enhance the inflammation through induction of chemokines production by epithelial cells (36) but also to promote the destruction of periodontal tissues by inducing T cells to produce TNF-α and RANKL (37). Although Th17 cells are the major source of IL-17A, ILC3s are also capable of producing IL-17A (5). Indeed, we observed ILC3s produced more IL-17A in periodontal ligament from periodontitis patients. IFN-γ is another pathogenic cytokine and is thought to promote periodontal inflammation (19). As expected, the percentage of IFN-γ–producing ILC1 and ILC3 cells was markedly increased in periodontitis. Together, these data suggest that both ILC1s and ILC3s promote periodontal inflammation during periodontitis through secreting inflammatory cytokines IFN-γ and IL-17A. Thus, targeting ILCs might represent a promising approach for treating periodontitis.

## Data Availability Statement

The original contributions presented in the study are included in the article/**Supplementary Material**. Further inquiries can be directed to the corresponding authors.

## Ethics Statement

The studies involving human participants were reviewed and approved by Ethical Review Board of Shanghai Stomatological Hospital, Fudan University. The patients/participants provided their written informed consent to participate in this study.

## Author Contributions

CL and JL collected the clinical samples, performed the experiments and analyzed the data. JP and YW collected the clinical samples. LS and YX conceived, designed and supervised the project. LS, YX, and JL wrote the manuscript. All authors contributed to the article and approved the submitted version.

## Funding

This study was supported by grant 202040495 (to YX) from Shanghai Municipal Health Commission, grant 81971487 (to LS) from the National Natural Science Foundation of China, grant 20ZR1430200 and 20142202300 (to LS) from Science and Technology Commission of Shanghai Municipality.

## Publisher’s Note

All claims expressed in this article are solely those of the authors and do not necessarily represent those of their affiliated organizations, or those of the publisher, the editors and the reviewers. Any product that may be evaluated in this article, or claim that may be made by its manufacturer, is not guaranteed or endorsed by the publisher.

## Conflict of Interest

The authors declare that the research was conducted in the absence of any commercial or financial relationships that could be construed as a potential conflict of interest.
