# Supplementary material for: ILC1s and ILC3s Exhibit Inflammatory Phenotype in Periodontal Ligament of Periodontitis Patients
Source: Front Immunol. 2021 Jul 26;12:708678. doi: 10.3389/fimmu.2021.708678 (PMC8350136; doi:10.3389/fimmu.2021.708678)
Supplement: Supplementary file 1 [file Presentation_1.pptx]

## Slide 1
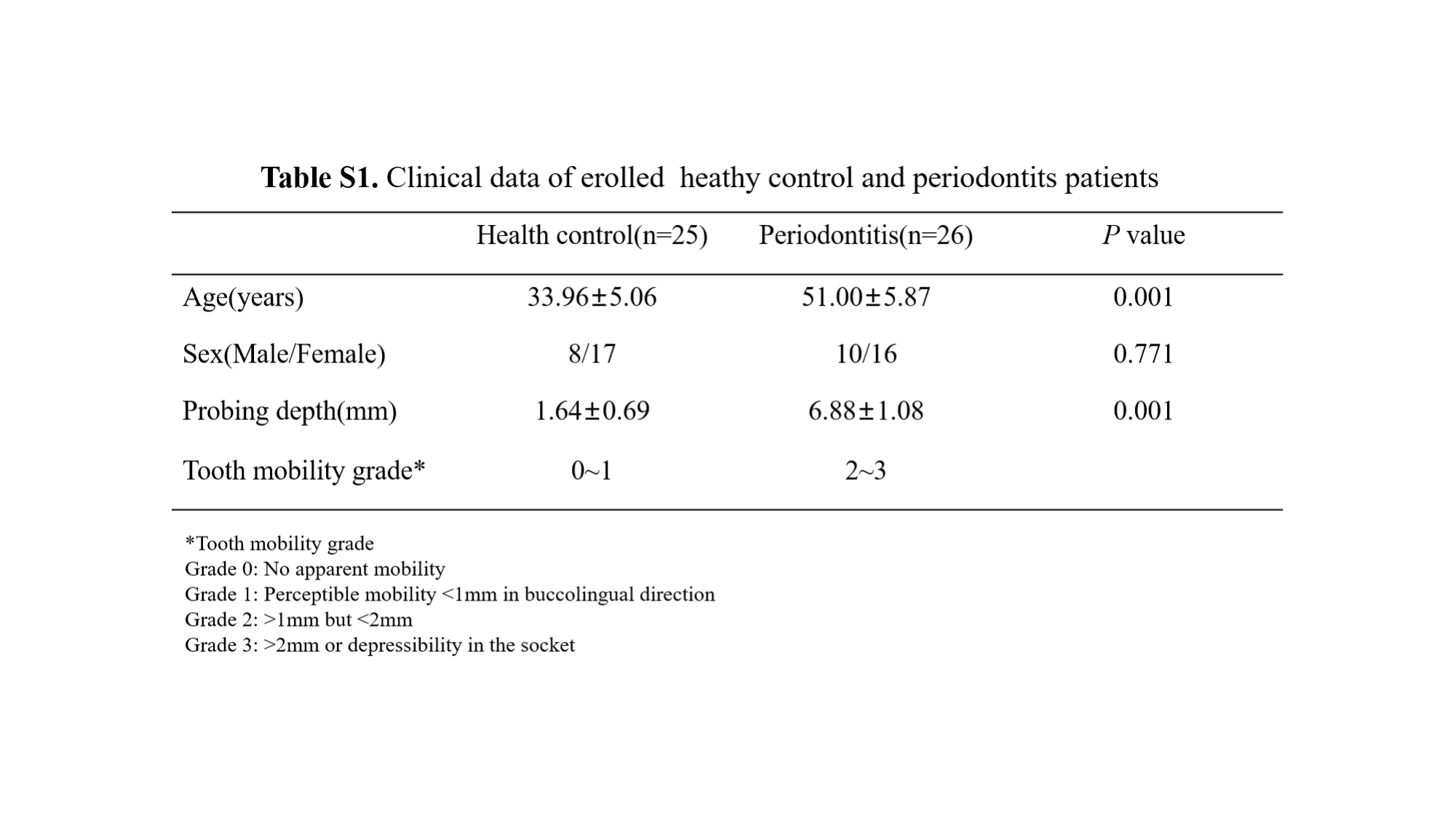

## Slide 2
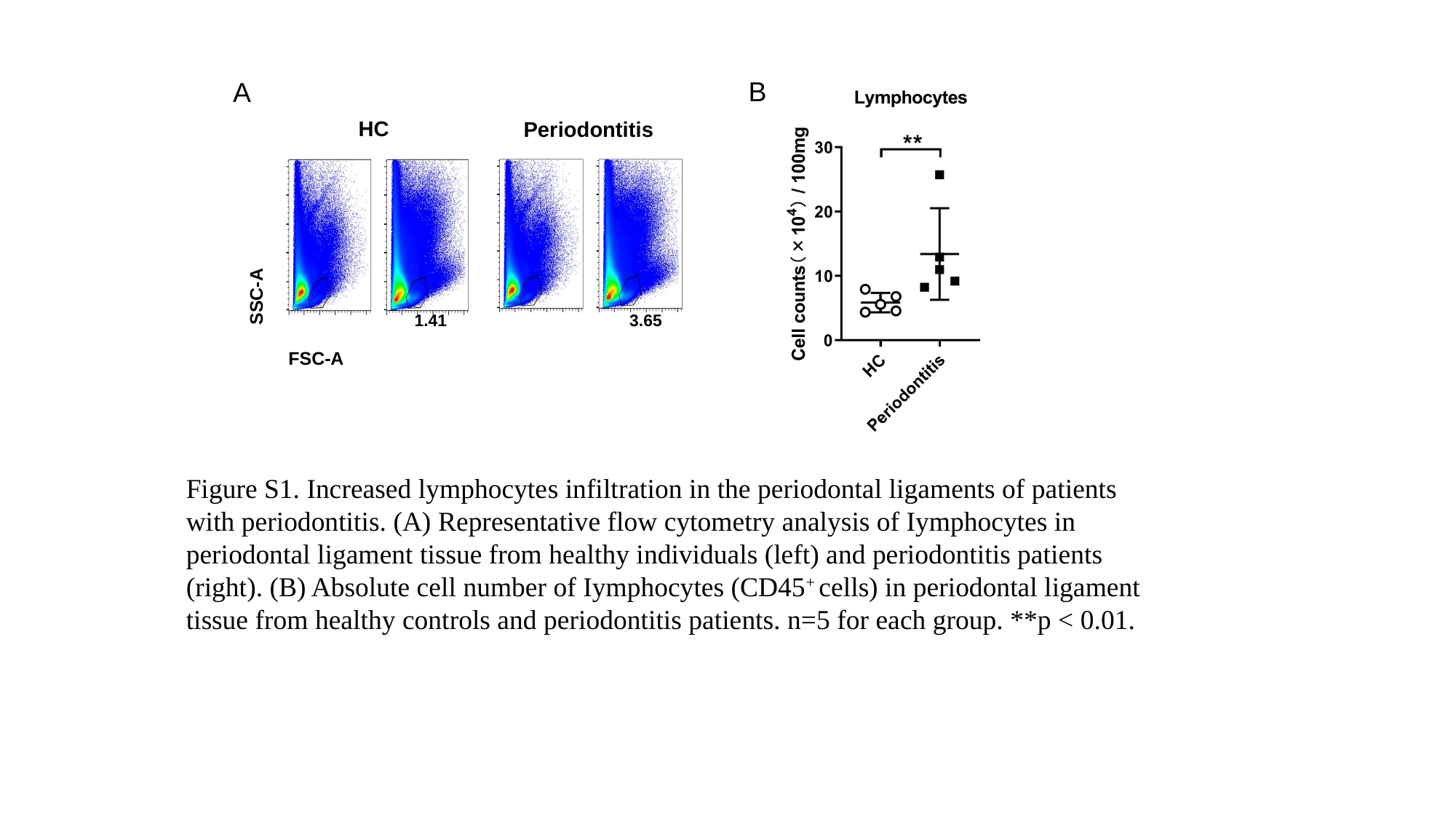

B
A
HC
Periodontitis
SSC-A
FSC-A
1.41
3.65
Figure S1. Increased lymphocytes infiltration in the periodontal ligaments of patients with periodontitis. (A) Representative flow cytometry analysis of Iymphocytes in periodontal ligament tissue from healthy individuals (left) and periodontitis patients (right). (B) Absolute cell number of Iymphocytes (CD45+ cells) in periodontal ligament tissue from healthy controls and periodontitis patients. n=5 for each group. **p < 0.01.

## Slide 3
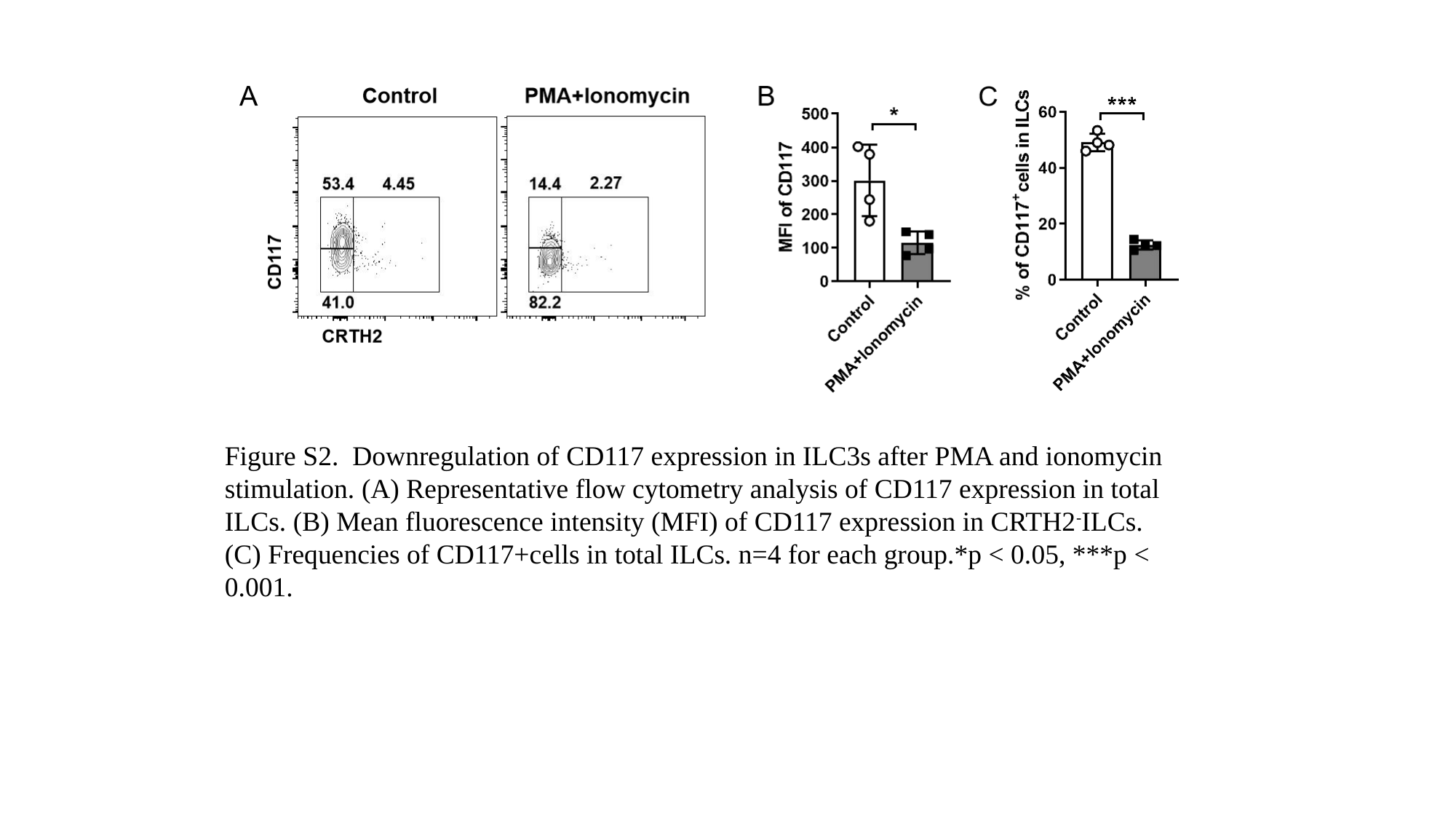

Figure S2. Downregulation of CD117 expression in ILC3s after PMA and ionomycin stimulation. (A) Representative flow cytometry analysis of CD117 expression in total ILCs. (B) Mean fluorescence intensity (MFI) of CD117 expression in CRTH2-ILCs. (C) Frequencies of CD117+cells in total ILCs. n=4 for each group.*p < 0.05, ***p < 0.001.
